# A discussion on significance indices for contingency tables under small sample sizes

**DOI:** 10.1371/journal.pone.0199102

**Published:** 2018-08-02

**Authors:** Natalia L. Oliveira, Carlos A. de B. Pereira, Marcio A. Diniz, Adriano Polpo

**Affiliations:** 1 Department of Statistics and Data Science, Carnegie Mellon Univesity, Pittsburgh, United States of America; 2 Department of Statistics, University of Sao Paulo, Sao Paulo, Brazil; 3 Department of Statistics, Federal University of Sao Carlos, Sao Carlos, Brazil; Politecnico di Torino, ITALY

## Abstract

Hypothesis testing in contingency tables is usually based on asymptotic results, thereby restricting its proper use to large samples. To study these tests in small samples, we consider the likelihood ratio test (LRT) and define an accurate index for the celebrated hypotheses of homogeneity, independence, and Hardy-Weinberg equilibrium. The aim is to understand the use of the asymptotic results of the frequentist Likelihood Ratio Test and the Bayesian FBST (Full Bayesian Significance Test) under small-sample scenarios. The proposed exact LRT p-value is used as a benchmark to understand the other indices. We perform analysis in different scenarios, considering different sample sizes and different table dimensions. The conditional Fisher’s exact test for 2 × 2 tables and the Barnard’s exact test are also discussed. The main message of this paper is that all indices have very similar behavior, except for Fisher and Barnard tests that has a discrete behavior. The most powerful test was the asymptotic p-value from the likelihood ratio test, suggesting that is a good alternative for small sample sizes.

## Introduction

We discuss indices for homogeneity, independence, and Hardy-Weinberg equilibrium hypotheses [1, 2] in contingency tables. We propose an exact evaluation of the Likelihood Ratio Test (LRT) as a benchmark significance index. Based on the work of [3], its idea is to evaluate the probability distribution of all possible tables on the sample space under the null hypothesis. Once the distribution for sampling contingency tables under the hypothesis is known, we are able to compute the exact distribution of the Likelihood Ratio Test (LRT) statistics. The main difficulty for this procedure is that it is computationally time-consuming, being only feasible for small sample sizes and/or for tables of small dimension.

The exact LRT p-value presented as a way to do exact inference. The aim is to compare the behavior of the frequentist LRT asymptotic p-value [4], the exact LRT p-value, the Fisher’s exact test p-value [5], the Chi-Square test asymptotic p-value [6, 7] and the Barnard’s exact test p-value [8–11]. These frequentist indices are also compared to the e-value from the Full Bayesian Significance Test (FBST) [12, 13]. It was considered the asymptotic e-value and its approximation (based on a Markov Chain Monte Carlo procedure) of the exact e-value. The choice of adding a Bayesian index to the comparison study originates from the known asymptotic relationship between the LRT and the FBST [14]. Moreover, the FBST and its e-value can be viewed as a Bayesian p-value counterpart, and therefore it is interesting to understand this Bayesian method when compared to frequentist methods. It is important to point out that we are mainly interested in the values of the indices, not in the acceptance or rejection of the hypothesis; that is, our focus is on the significance test, which consists of the evaluation of the p-(e-)values. In an applied setting, the researcher can, based on the indices, make his/her decision about his/her application. We are not interested in comparing the values of the indices with some fixed significance value (generally 5%) to decide the if the hypothesis should be accepted or rejected. With this goal in mind, all significance indices considered here are in agreement with the ASA’s statement on significance indices [15].

From a historical perspective, hypothesis testing has been the most widely used statistical tool in many fields of science [16–18]. For categorical data, [19] discusses some exact procedures to perform inference and [20] presents methodological procedures for hypothesis testing for contingency tables. Tests for homogeneity hypothesis in contingency tables have been compared by [21], who compared the conditional and unconditional, and by [22], who compares, under an asymptotic perspective, two tests for equality of two proportions considering Goodman’s *Y*^2^ and *χ*^2^ statistics. Regarding tests for the independence of two classifiers in contingency tables, [23] presents an algorithm for finding the exact permutation significance level for *r* × *c* contingency tables. [24], studies a simple way to compare two correlated proportions. More recently, [25] presents the exact likelihood ratio test for equality of two normal populations, and [26] discuss exact unconditional tests for homogeneity hypothesis in 2 × 2 tables.

One important aspect that differentiates the tests procedures is how each one deals with the elimination of the nuisance parameter. Basu [27] lists several methods but focuses on marginalization and conditioning. He defines *marginalization* as every procedure that replaces the observed sample *x* by the observed value of a suitable statistic *T*(*x*) = *t*. Therefore, instead of working with the original experiment ℰ and data *x*, one should use the *marginal* experiment ${ℰ}_{T}$ and the recorded value *T*(*x*) since the *marginal* statistical model would depend only on the parameter of interest. To justify these procedures, Basu adds that researchers usually recur to invariance or partial sufficiency arguments.

By *conditioning*, Basu defines methods of elimination that also consist of choosing a suitable statistic, but such that the conditional distribution of the observed sample, *x*, given the observed value of the statistic depends on the full parameter space only through the parameter of interest. Another commonly used approach that Basu describes is the one he calls *maximization*. In this case the nuisance parameter is eliminated from the risk function by some sort of maximization (or minimax) principle or directly from the likelihood, usually maximizing it with respect the nuisance parameters.

A final important strategy mentioned by Basu is the one he called *Bayesian solution*. In this case, one should derive the full posterior and integrate out the nuisance parameters, obtaining the posterior marginal distribution necessary to perform the required statistical inference. It is important to point out that the FBST does not follow this Bayesian strategy, since its evidence value is computed considering the full posterior. The proposed exact LRT p-value is based on the idea of integrating out the nuisance parameter, which is in some way related to Basu’s *Bayesian solution* [26]. The methods for elimination of nuisance parameters, *maximization* and *Bayesian solution* can be considered as unconditional methods.

The Likelihood Ratio Test (LRT) asymptotic p-value [28], the Chi-Square test asymptotic p-value [29], Fisher’s homogeneity exact test [29, 30], Barnard’s exact test [8], and the Full Bayesian Significance Test (FBST) asymptotic and exact e-value [12, 13] are presented in detail for the case of 2 × 2 contingency tables considering homogeneity hypothesis (Section 1.1). The theoretical results for homogeneity and independence hypotheses for tables of any dimension and Hardy-Weinberg equilibrium hypothesis are presented in sections 1.2, 1.3 and 1.4.

We study the relationship between indices in Section 2.1. [14] perform a similar study, however they consider continuous random variables using the e-value and the LRT p-value and show that these indices share an asymptotic relationship. In our case, the asymptotic LRT p-value, the exact LRT p-value and the Chi-Square p-value have similar behavior, including in small sample size scenarios. Both Fisher’s exact test and Barnard’s exact test have a discrete behavior for their p-values, being more clear for the Barnard’s exact test p-value. All tests are unconditional tests, except for the Fisher one, that is a conditional test. It is important to draw attention to the fact that the present results are not based on a simulation study, we compute the indices for all possible tables in the sample space.

In addition to our focus on the study of significance indices, we also provide, for the frequentist indices, a study of the power functions to compare the tests considering the homogeneity hypothesis (2 × 2 tables) and Hardy-Weinberg equilibrium hypothesis (Section 2.2). The Fisher’s exact test was the least powerful, followed by the Barnard’s exact test, Chi-Square test, the exact LRT and the asymptotic LRT, the most powerful one. We did not evaluate the power function for the FBST; firstly, because it is not the aim of the Bayesian paradigm, and secondly, to do so, it would be necessary to define a decision rule for the FBST, which is not in the scope of this paper. We also note that, under the hull hypothesis, considering the significance level 5%, all frequentist indices achieved 5% rejection as expected.

## 1 Methods

### 1.1 Homogeneity test for 2 × 2 contingency tables

Let *X*_1_ and *X*_2_ be two random variables, representing the rows (1 and 2) of Table 1, *x*_11_ and *x*_21_ being their observed values, and *n*_1⋅_ and *n*_2⋅_ fixed sample sizes. Consider the distributions of *X*_1_ as Binomial(*n*_1⋅_, *θ*_11_) and *X*_2_ a Binomial(*n*_2⋅_, *θ*_21_) for describing the chances of a subject belong to category (column) *C*_1_ in two distinct populations. Both populations are partitioned into two categories (columns) *C*_1_ and *C*_2_ and the objective is to test homogeneity among the two unknown population frequencies, ***H***: *θ*_11_ = *θ*_21_ = *θ*. This hypothesis is geometrically represented by a diagonal line of the unit square.

**Table 1:** Contingency table 2 × 2.

row\column	1	2	total
1	*x*_11_	*x*_12_	*n*_1⋅_
2	*x*_21_	*x*_22_	*n*_2⋅_

*n*_*i*⋅_ = *x*_*i*1_ + *x*_*i*2_, *i* = 1, 2.

The likelihood function is specified by

$$\begin{array}{c} L\left(\theta_{11},\theta_{21}|x_{11},x_{21},n_{1\cdot },n_{2\cdot }\right)=\frac{n_{1\cdot }!n_{2\cdot }!}{x_{11}!x_{21}!x_{12}!x_{22}!}\theta_{11}^{x_{11}}\theta_{21}^{x_{21}}{\left(1-\theta_{11}\right)}^{x_{12}}{\left(1-\theta_{21}\right)}^{x_{22}}, \end{array}$$ (1)

where 0 ≤ *θ*_*i*1_ ≤ 1, *i* = 1, 2. Under ***H***, the likelihood function simplifies to

$$\begin{array}{c} L\left(\theta |x_{11},x_{21},n_{1\cdot },n_{2\cdot },H\right)=\frac{n_{1\cdot }!n_{2\cdot }!}{x_{11}!x_{21}!x_{12}!x_{22}!}\theta^{x_{11}+x_{21}}{\left(1-\theta \right)}^{x_{12}+x_{22}},0\leq \theta \leq 1, \end{array}$$ (2)

and the LRT test statistics is:

$$\begin{array}{lll} \lambda \left(x_{11},x_{21}\right) & = & \frac{\underset{\theta \in \Theta_{H}}{\text{sup}}L\left(\theta_{11},\theta_{21}|x_{11},x_{21},n_{1\cdot },n_{2\cdot }\right)}{\underset{\theta \in \Theta }{\text{sup}}L\left(\theta_{11},\theta_{21}|x_{11},x_{21},n_{1\cdot },n_{2\cdot }\right)}\\ & = & \frac{{\left(\frac{x_{11}+x_{21}}{n_{1\cdot }+n_{2\cdot }}\right)}^{x_{11}+x_{21}}{\left(\frac{x_{12}+x_{22}}{n_{1\cdot }+n_{2\cdot }}\right)}^{x_{12}+x_{22}}}{{\left(\frac{x_{11}}{n_{1\cdot }}\right)}^{x_{11}}{\left(\frac{x_{12}}{n_{1\cdot }}\right)}^{x_{12}}{\left(\frac{x_{21}}{n_{2\cdot }}\right)}^{x_{21}}{\left(\frac{x_{22}}{n_{2\cdot }}\right)}^{x_{22}}}, \end{array}$$ (3)

in which Θ_***H***_ is the parametric set defined by the hypothesis.

• **Exact LRT p-value:**

To define this p-value, we use the predictive distributions of *X*_1_ and *X*_2_ before any data were observed. The proposed p-value is an alternative way to calculate an exact p-value for the LRT. The goal is to find a distribution for the contingency table under ***H*** that is not a function on *θ*. We consider *θ* a nuisance parameter in the likelihood function in (2) and integrate it over *θ* in order to eliminate it, as suggested by [27]. The idea is to incorporate the concept of the *Bayesian solution* nuisance parameter elimination approach but in a frequentist setting, which means using the likelihood function instead of a posterior distribution. That is,

$$\begin{array}{ll} h(x_{11},x_{21}) & =\int_{0}^{1}L(\theta |x_{11},x_{21},n_{1\cdot },n_{2\cdot },H)\text{d}\theta \\ & =\frac{n_{1\cdot }!n_{2\cdot }!}{x_{11}!x_{21}!x_{12}!x_{22}!}\int_{0}^{1}\theta^{x_{11}+x_{21}}{\left(1-\theta \right)}^{x_{12}+x_{22}}\text{d}\theta \\ & =\left(\begin{array}{c} n_{1\cdot }\\ x_{11} \end{array}\right)\left(\begin{array}{c} n_{2\cdot }\\ x_{21} \end{array}\right)\frac{(x_{11}+x_{21})!(x_{12}+x_{22})!}{(n_{1\cdot }+n_{2\cdot }+1)!}\\ & =\frac{\left(\begin{array}{c} n_{1\cdot }\\ x_{11} \end{array}\right)\left(\begin{array}{c} n_{2\cdot }\\ x_{21} \end{array}\right)}{\left(\begin{array}{c} n_{1\cdot }+n_{2\cdot }\\ x_{11}+x_{21} \end{array}\right)}\frac{1}{\left(n_{1\cdot }+n_{2\cdot }+1\right)}. \end{array}$$ (4)

To obtain the probability function Pr(*X*_1_ = *x*_11_, *X*_2_ = *x*_21_ ∣ ***H***), one needs to find a normalization constant.

$$\begin{array}{c} \text{Pr}\left(X_{1}=x_{11},X_{2}=x_{21}|H\right)=\frac{h(x_{11},x_{21})}{\sum_{i=0}^{n_{1\cdot }}\sum_{j=0}^{n_{2\cdot }}h\left(i,j\right)}. \end{array}$$ (5)

Note that to calculate (5), we evaluate *h*(⋅, ⋅) for all possible tables. In the case of a homogeneity hypothesis for 2 × 2 contingency tables, $\sum_{i=0}^{n_{1\cdot }}\sum_{j=0}^{n_{2\cdot }}h(i,j)=1$. We present the table’s probability in terms of this sum to obtain a general formula for all hypotheses and table dimensions considered here, since in other scenarios this quantity does not sum up to 1 (for example, the sum of *h* for all possible 2 × 2 tables considering independence hypothesis with *n* = 2 is 2304). The exact p-value calculation follows directly from the test statistic distribution:

$$\begin{array}{ccc} \text{p-value} & = & \text{Pr}(\lambda \left(X_{1},X_{2}\right)\leq \lambda \left(x_{11},x_{21}\right)|H)\\ & = & \sum_{(i,j)\in R}\text{Pr}\left(X_{1}=i,X_{2}=j|H\right), \end{array}$$ (6)

in which ***R*** is the set of all pairs (*i*, *j*) such that λ(*i*, *j*) ≤ λ(*x*_11_, *x*_21_), and λ(*x*_11_, *x*_21_) is the observed test statistic, as in (3).

• **Barnard’s Exact Test:**

Consider that *n*_1⋅_ and *n*_2⋅_ are fixed in Table 1. The random variables *X*_1_ and *X*_2_ are independent Binomial distribution with parameters *θ*_11_ and *θ*_21_. The probability of a sample {*x*_11_, *x*_21_} be drawn is

$$\begin{array}{c} \text{Pr}\left(X_{1}=x_{11},X_{2}=x_{21}\right)=\frac{n_{1\cdot }!}{x_{11}!x_{12}!}\theta_{11}^{x_{11}}{\left(1-\theta_{11}\right)}^{x_{12}}\frac{n_{2\cdot }!}{x_{21}!x_{22}!}\theta_{21}^{x_{21}}{\left(1-\theta_{21}\right)}^{x_{22}}, \end{array}$$ (7)

and, under hypothesis ***H***,

$$\begin{array}{c} \text{Pr}\left(X_{1}=x_{11},X_{2}=x_{21}|H\right)=\frac{n_{1\cdot }!n_{2\cdot }!}{x_{11}!x_{12}!x_{21}!x_{22}!}\theta^{x_{11}+x_{12}}{\left(1-\theta \right)}^{x_{12}+x_{22}}. \end{array}$$ (8)

We define the critical region as ***R*** = {λ(*X*_1_, *X*_2_) ≤ λ(*x*_11_, *x*_21_)}, then the Barnard’s exact p-value is obtained by

$$\begin{array}{c} \text{p-value}=\underset{0\leq \theta \leq 1}{\text{max}}\sum_{R}\frac{n_{1\cdot }!n_{2\cdot }!}{x_{11}!x_{12}!x_{21}!x_{22}!}\theta^{x_{11}+x_{12}}{\left(1-\theta \right)}^{x_{12}+x_{22}}. \end{array}$$ (9)

That is, the Barnard’s exact test consider the p-values for all possible points of the parameter space under ***H***, and takes the maximum p-value. In this test, the chosen approach for nuisance parameter elimination among the ones presented by Basu is *maximization*.

• **Full Bayesian Significance Test:**

The Bayesian approach considered is based on the FBST (Full Bayesian Significance Test) [12, 13].

**Definition 1** *Let π*(*θ* ∣ ***x***) *be the posterior density function of θ given the observed sample and* $T(x)=\{\theta \in \Theta :\pi (\theta |x)\geq {\text{sup}}_{\theta \in \Theta_{H}}\pi (\theta |x)\}$. *The supporting evidence measure for the hypothesis θ* ∈ Θ_***H***_ is defined as *Ev*(Θ_***H***_, ***x***) = 1 − Pr(*θ* ∈ *T*(***x***) ∣ ***x***).

Consider that, *a priori*, *θ*_11_ and *θ*_21_ are independent and both follow a Uniform(0, 1) distribution. The choice of uniforms priors is to avoid a subjective prior to have a fair comparison with frequentist indices. Recall that *X*_1_ and *X*_2_ given *θ*_11_ and *θ*_21_ are Binomial distributed. Hence, the posterior distributions for *θ*_11_ and *θ*_21_ are independent Beta(*x*_11_ + 1, *n*_1⋅_ − *x*_11_ + 1) and Beta(*x*_21_ + 1, *n*_2⋅_ − *x*_21_ + 1). Under the hypothesis ***H***, the posterior distribution is

$$\begin{array}{c} \pi \left(\theta |x_{11},x_{21},n_{1\cdot },n_{2\cdot },H\right)=\frac{\theta^{x_{11}+x_{21}}{\left(1-\theta \right)}^{x_{12}+x_{22}}}{B\left(x_{11}+1,x_{12}+1\right)B\left(x_{21}+1,x_{22}+1\right)} \end{array}$$ (10)

and by maximizing it in *θ* we obtain sup_*θ*∈(0,1)_ *π*(*θ* ∣ *x*_11_, *x*_21_, *n*_1⋅_, *n*_2⋅_, ***H***), where ℬ(⋅,⋅) is the Beta function. Since *x*_11_, *x*_21_, *n*_1⋅_ and *n*_2⋅_ are integers,

$$\begin{array}{l} \pi \left(\theta |x_{11},x_{21},n_{1\cdot },n_{2\cdot },H\right)=\\ \left(\begin{array}{c} n_{1\cdot }\\ x_{11} \end{array}\right)\left(\begin{array}{c} n_{2\cdot }\\ x_{21} \end{array}\right)\left(n_{1\cdot }+1\right)\left(n_{2\cdot }+1\right)\theta^{x_{11}+x_{21}}{\left(1-\theta \right)}^{x_{12}+x_{22}}, \end{array}$$ (11)

$$\begin{array}{l} \underset{\theta \in \left(0,1\right)}{\text{sup}}\pi \left(\theta |x_{11},x_{21},n_{1\cdot },n_{2\cdot },H\right)=\\ \frac{\left(n_{1\cdot }+1\right)!\left(n_{2\cdot }+1\right)!}{x_{11}!x_{21}!x_{12}!x_{22}!}{\left(\frac{x_{11}+x_{21}}{n_{1\cdot }+n_{2\cdot }}\right)}^{x_{11}+x_{21}}{\left(\frac{x_{12}+x_{22}}{n_{1\cdot }+n_{2\cdot }}\right)}^{x_{12}+x_{22}}, \end{array}$$ (12)

the hypothesis’ tangent set, *T*, is

$$\begin{array}{c} T\left(x_{11},x_{21},n_{1\cdot },n_{2\cdot }\right)=\{\left(\theta_{11},\theta_{21}\right)\in \left(0,1\right)\times \left(0,1\right):\\ \pi \left(\theta_{11},\theta_{21}|x_{11},x_{21},n_{1\cdot },n_{2\cdot }\right)\geq \underset{\theta \in (0,1)}{\text{sup}}\pi \left(\theta |x_{11},x_{21},n_{1\cdot },n_{2\cdot },H\right)\}, \end{array}$$ (13)

and

$$\begin{array}{c} \text{e-value}=1-\text{Pr}[\theta \in T\left(x_{11},x_{21},n_{1\cdot },n_{2\cdot }\right)]. \end{array}$$ (14)

To calculate the approximate e-value, we use the following algorithm:

1. A random sample of size *k* is generated from posterior distribution of *θ*_11_, *θ*_21_, obtaining $\left\{\theta_{x_{11}1},\theta_{x_{21}1}\},\ldots ,\{\theta_{x_{11}k},\theta_{x_{21}k}\right\}$ .
2. The e-value is calculated by
  $$\begin{array}{c} \;1-\frac{1}{k}\sum_{i=1}^{k}I(\pi \left(\theta_{x_{11}i},\theta_{x_{21}i}|x_{11},x_{21},n_{1\cdot },n_{2\cdot }\right)\geq \underset{\theta \in (0,1)}{\text{sup}}\pi \left(\theta |x_{11},x_{21},n_{1\cdot },n_{2\cdot }\right)), \end{array}$$
  in which *I*(*A*) is the indicator function of set *A*.

• **Other indices:**

For the LRT, the statistic −2 ln[λ(*X*_1_, *X*_2_)] has asymptotically a chi-square distribution with 1 degree of freedom, which is *dim*(Θ) − *dim*(Θ_***H***_) [28]. The FBST uses the same statistic, however its asymptotic distribution is a chi-square with 2 degrees of freedom [13], which is *dim*(Θ). For the chi-square test and the Fisher’s exact test for homogeneity see [29].

### 1.2 Homogeneity hypothesis for *ℓ* × *c* contingency tables

Let *X*_*i*_, *i* = 1, …, *ℓ*, be random variables that are represented by the rows of Table 2 and *n*_1⋅_, *n*_2⋅_, …, *n*_*ℓ*⋅_ are known constants.

**Table 2:** Contingency table *ℓ* × *c*.

row\column	1	2	⋯	*c*	total
1	*x*_11_	*x*_12_		*x*_1*c*_	*n*_1⋅_
2	*x*_21_	*x*_22_		*x*_2*c*_	*n*_2⋅_
⋮			⋱	⋮	⋮
*ℓ*	*x*_*ℓ*1_	*x*_*ℓ*2_	⋯	*x*_*ℓc*_	*n*_*ℓ*⋅_
total	*n*_⋅1_	*n*_⋅2_	⋯	*n*_⋅*c*_	*n*_⋅⋅_

$n_{i\cdot }=\sum_{j=1}^{c}x_{ij}$, *i* = 1, …, *ℓ*;

$n_{\cdot j}=\sum_{i=1}^{\ell }x_{ij}$, *j* = 1, …, *c*; and

$n_{\cdot \cdot }=\sum_{i=1}^{\ell }\sum_{j=1}^{c}x_{ij}$.

Assuming that *X*_*i*_, *i* = 1, …, *ℓ*, follows a Multinomial(*n*_*i*⋅_, *θ*_*i*1_, …, *θ*_*ic*_) distribution, we are interested in testing if their distributions are homogeneous with respect to categories (columns) *C*_*j*_, *j* = 1, …, *c*. That is,

$$\begin{array}{c} H:\{\begin{array}{ccc} \theta_{1} & = & \theta_{11}=\theta_{21}=\cdots =\theta_{\ell 1},\\ \theta_{2} & = & \theta_{12}=\theta_{22}=\cdots =\theta_{\ell 2},\\ & \vdots & \\ \theta_{c-1} & = & \theta_{1(c-1)}=\theta_{2(c-1)}=\cdots =\theta_{\ell (c-1)}, \end{array} \end{array}$$

in which $\theta_{c}=1-\sum_{k=1}^{c-1}\theta_{k}$, 0 ≤ *θ*_*k*_ ≤ 1, ∀*k* = 1, …, *c*.

Let ***x*** be all observed values presented in Table 2 and ***θ*** all the parameters. The likelihood function is

$$\begin{array}{c} L\left(\theta |x\right)=\left[\prod_{i=1}^{\ell }n_{i\cdot }!/\prod_{i=1}^{\ell }\prod_{j=1}^{c}x_{ij}!\right]\prod_{i=1}^{\ell }\prod_{j=1}^{c}\theta_{ij}^{x_{ij}}, \end{array}$$ (15)

and under the hypothesis ***H***,

$$\begin{array}{c} L\left(\theta |x,H\right)=[\prod_{i=1}^{\ell }n_{i\cdot }!/\prod_{i=1}^{\ell }\prod_{j=1}^{c}x_{ij}!]\prod_{j=1}^{c}\theta_{j}^{n_{\cdot j}}. \end{array}$$ (16)

The LRT λ statistic is

$$\begin{array}{c} \lambda \left(x\right)=\prod_{j=1}^{c}{\left(\frac{n_{\cdot j}}{n_{\cdot \cdot }}\right)}^{n_{\cdot j}}/\prod_{i=1}^{l}\prod_{j=1}^{c}{\left(\frac{x_{ij}}{n_{i\cdot }}\right)}^{x_{ij}}. \end{array}$$ (17)

• **Exact LRT p-value:**

To obtain the exact LRT p-value, we need the function *h*(***x***). In this scenario,

$$\begin{array}{c} h\left(x\right)=\prod_{i=1}^{\ell }n_{i\cdot }!\prod_{j=1}^{c}n_{\cdot j}!/[(\prod_{i=1}^{\ell }\prod_{j=1}^{c}x_{ij}!)\left(n_{\cdot \cdot }+c-1\right)!], \end{array}$$ (18)

and the p-value’s calculation follows as in Subsection 1.1.

• **FBST:**

Assuming a Dirichlet(1, 1, …, 1) prior for {*θ*_*i*1_, …, *θ*_*ic*_}, and since ***X***_***i***_ follows a Multinomial(*n*_*i*_, *θ*_*i*1_, …, *θ*_*ic*_) distribution, then the posterior distribution is a Dirichlet(*x*_*i*1_ + 1, *x*_*i*2_ + 1, …, *x*_*ic*_ + 1), *i* = 1, …, *ℓ*.

In this setting,

$$\begin{array}{c} \underset{\theta \in \Theta_{H}}{\text{sup}}\pi \left(\theta |x\right)=\prod_{i=1}^{l}\left(n_{i\cdot }+c-1\right)!\prod_{j=1}^{c}{\left(\frac{n_{\cdot j}}{n_{\cdot \cdot }}\right)}^{n_{\cdot j}}/\prod_{i=1}^{l}\prod_{j=1}^{c}x_{ij}!, \end{array}$$ (19)

and we can obtain the e-value from Definition 1.

• **Other indices:**

Both asymptotic LRT p-value and asymptotic e-value are calculated as Pr[−2 ln(λ(***X***)) ≤ −2 ln(λ(***x***))], but while the LRT considers that this statistic follows a $X^{2}$ distribution with (*ℓ* − 1)(*c* − 1) degrees of freedom, the FBST considers that it follows a $X^{2}$ distribution with *ℓ*(*c* − 1) degrees of freedom. The Chi-Square homogeneity test is also obtained.

### 1.3 Independence hypothesis for *ℓ* × *c* contingency tables

Consider that *θ*_*ij*_ is the probability of observing a sample in the cell at row *i* and column *j*, *θ*_*i*⋅_ is the probability of observing a sample in row *i*, *θ*_⋅*j*_ is the probability of observing a sample in column *j*, 0 ≤ *θ*_*ij*_ ≤ 1, 0 ≤ *θ*_*i*⋅_ ≤ 1, 0 ≤ *θ*_⋅*j*_ ≤ 1, *i* = 1, …, *ℓ*, *j* = 1, …, *c*, $\sum_{i=1}^{\ell }\sum_{j=1}^{c}\theta_{ij}=1$, $\sum_{i=1}^{\ell }\theta_{i\cdot }=1$, and $\sum_{j=1}^{c}\theta_{\cdot j}=1$.

**Fig 1:**
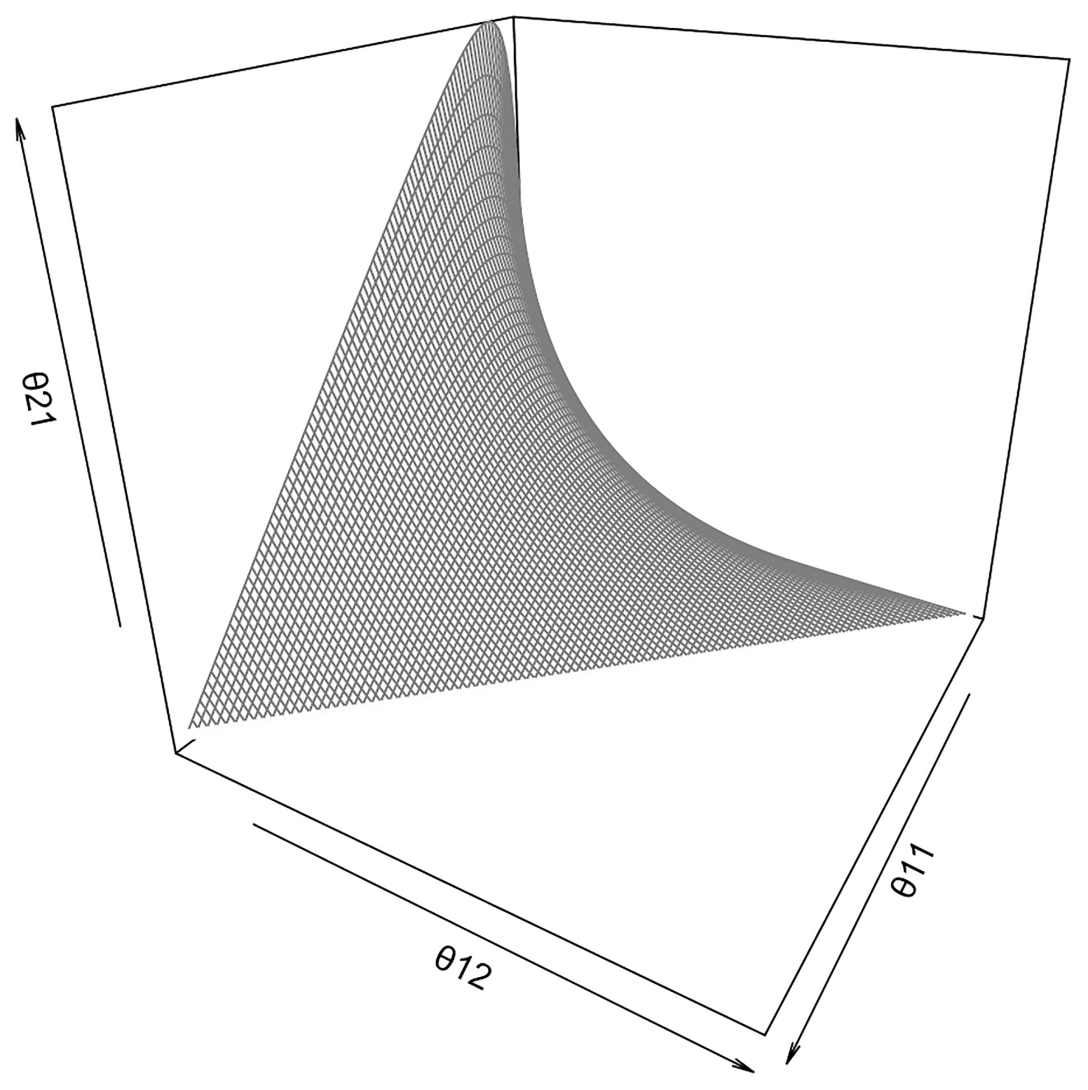
Geometric representation of the independence hypothesis (gray surface) for 2 × 2 tables. The parametric space is the three-dimensional simplex (regular tetrahedron).

For the independence hypothesis, our interest is to test ***H***: *θ*_*ij*_ = *θ*_*i*⋅_ × *θ*_⋅*j*_, ∀*i*, *j*. For the case of 2 × 2 table, the independence hypothesis is geometrically represented as Fig 1.

Considering that *n*_⋅⋅_ is known, we assume that the outcomes of Table 2 follow a Multinomial(*n*.., ***θ***) distribution, ***θ*** = {*θ*_11_, …, *θ*_1(*c*−1)_, …, *θ*_*ℓ*1_, …, *θ*_*ℓ*(*c*−1)_}, and $\theta_{ic}=1-\sum_{j=1}^{c-1}\theta_{ij}$, *i* = 1, …, *ℓ*. The likelihood function is

$$\begin{array}{c} L\left(\theta |x\right)=[n_{\cdot \cdot }!/\prod_{i=1}^{\ell }\prod_{j=1}^{c}x_{ij}!]\prod_{i=1}^{\ell }\prod_{j=1}^{c}\theta_{ij}^{x_{ij}}. \end{array}$$ (20)

The likelihood function under ***H*** is

$$\begin{array}{c} L\left(\theta |x,H\right)=[n_{\cdot \cdot }!/\prod_{i=1}^{\ell }\prod_{j=1}^{c}x_{ij}!]\prod_{i=1}^{\ell }\theta_{i\cdot }^{n_{i\cdot }}\prod_{j=1}^{c}\theta_{\cdot j}^{n_{\cdot j}}, \end{array}$$ (21)

and the LRT λ statistic is

$$\begin{array}{c} \lambda \left(x\right)=\prod_{i=1}^{l}{\left(\frac{n_{i\cdot }}{n_{\cdot \cdot }}\right)}^{n_{i\cdot }}\prod_{j=1}^{c}{\left(\frac{n_{\cdot j}}{n_{\cdot \cdot }}\right)}^{n_{\cdot j}}/\prod_{i=1}^{l}\prod_{j=1}^{c}{\left(\frac{x_{ij}}{n_{\cdot \cdot }}\right)}^{x_{ij}}. \end{array}$$ (22)

• **Exact LRT p-value:**

As shown in Subsection 1.1, this p-value is obtained the same way but with a different *h*(***x***). In this case,

$$\begin{array}{c} h\left(x\right)=n_{\cdot \cdot }!\left(n_{\cdot \cdot }+\ell \right)!\left(n_{\cdot \cdot }+c\right)!/[\prod_{i=1}^{\ell }\prod_{j=1}^{c}x_{ij}!\prod_{i=1}^{\ell }n_{i\cdot }!\prod_{j=1}^{c}n_{\cdot j}!]. \end{array}$$ (23)

• **FBST:**

Assuming a Dirichlet(1, …, 1) as prior distribution for ***θ*** and that the outcomes of Table 2 follow a Multinomial(*n*, *θ*_11_, …, *θ*_1*c*_, …, *θ*_*ℓ*1_, …, *θ*_*ℓc*_) distribution, then the posterior distribution is a Dirichlet(*x*_11_ + 1, …, *x*_1*c*_ + 1, …, *x*_*ℓ*1_ + 1, …, *x*_*ℓc*1_ + 1). The e-value is obtained from Definition 1 and

$$\begin{array}{c} \underset{\theta \in \Theta_{H}}{\text{sup}}\pi \left(\theta |x\right)=\left(n+lc-1\right)!\prod_{i=1}^{l}{\left(\frac{n_{i\cdot }}{n}\right)}^{n_{i\cdot }}\prod_{j=1}^{c}{\left(\frac{n_{\cdot j}}{n}\right)}^{n_{\cdot j}}/\left[\prod_{i=1}^{l}\prod_{j=1}^{c}x_{ij}!\right]. \end{array}$$ (24)

• **Other indices:**

We obtained the asymptotic LRT p-value and e-value, considering that −2*ln*(λ(***X***)) follows a $X^{2}$ distribution with (*ℓ* − 1)(*c* − 1) and (*ℓc* − 1) degrees of freedom. We also obtained the p-value for the Chi-Square independence test.

### 1.4 Hardy-Weinberg equilibrium

An individual’s genotype is formed by a combination of alleles. If there are two possible alleles for one characteristic (say *A* and *a*), the possible genotypes are *AA*, *Aa* or *aa*. Considering a few premises true [31], the principle says that the allele probability in a population does not change from generation to generation. It is a fundamental principle for the Mendelian mating allelic model. If the probabilities of alleles are *θ* and 1 − *θ*, the expected genotype probabilities are (*θ*^2^, 2*θ*(1 − *θ*), (1 − *θ*)^2^) 0 ≤ *θ* ≤ 1.

**Fig 2:**
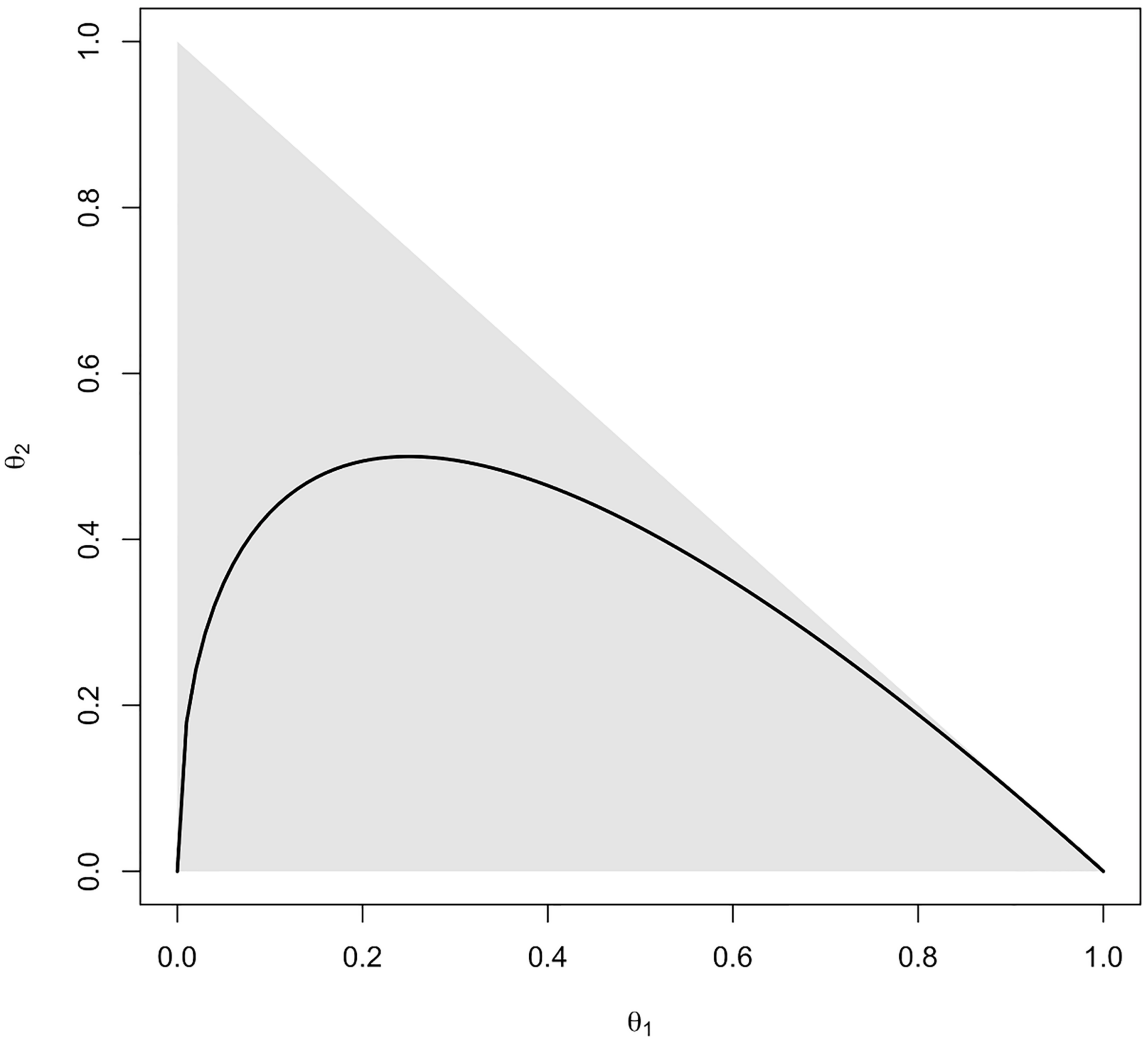
Geometric representation of the Hardy-Weinberg equilibrium hypothesis (black line), and the parametric space (gray shading).

Considering the Hardy-Weinberg equilibrium, the aim is to verify if a population follows these genotypes proportions. Therefore, the equilibrium hypothesis is

$$\begin{array}{c} H:\{\begin{array}{ccc} \theta_{1} & = & \theta^{2},\\ \theta_{2} & = & 2\theta (1-\theta ),\\ \theta_{3} & = & {\left(1-\theta \right)}^{2}, \end{array} \end{array}$$

in which *θ*_1_, *θ*_2_, *θ*_3_ are the proportions of AA, Aa, and aa, respectively. This hypothesis is geometrically represented in Fig 2.

Let *X* be a random vector. Table 3 represents the genotype frequencies for the population in question. Considering *n* known, we assume that *X* follows a Trinomial(*n*, *θ*_1_, *θ*_2_, *θ*_3_) distribution. The likelihood function for this model is

$$\begin{array}{c} L\left(\theta |x\right)=[\frac{n!}{x_{1}!x_{2}!x_{3}!}]\theta_{1}^{x_{1}}\theta_{2}^{x_{2}}\theta_{3}^{x_{3}}, \end{array}$$ (25)

in which ***x*** = {*x*_1_, *x*_2_, *x*_3_}, *θ*_1_ + *θ*_2_ + *θ*_3_ = 1 and *θ*_*i*_ > 0, *i* = 1, 2, 3. Under the hypothesis ***H***,

$$\begin{array}{c} L\left(\theta |x,H\right)=[\frac{n!}{x_{1}!x_{2}!x_{3}!}]2^{x_{2}}\theta^{2x_{1}+x_{2}}{\left(1-\theta \right)}^{2x_{3}+x_{2}},0\leq \theta \leq 1. \end{array}$$ (26)

**Table 3:** Genotype frequency.

	*AA*	*Aa*	*aa*	total
*X*	*x*_1_	*x*_2_	*x*_3_	*n*

*n* = *x*_1_ + *x*_2_ + *x*3.

The maximum likelihood estimator for *θ* under ***H*** is $\hat{\theta }=(2x_{1}+x_{2})/(2n)$ and the LRT λ statistic is

$$\begin{array}{c} \lambda \left(x\right)=\frac{2^{x_{2}}{\hat{\theta }}^{2x_{1}+x_{2}}{\left(1-\hat{\theta }\right)}^{2x_{3}+x_{2}}}{{\left(\frac{x_{1}}{n}\right)}^{x_{1}}{\left(\frac{x_{2}}{n}\right)}^{x_{2}}{\left(\frac{x_{3}}{n}\right)}^{x_{3}}}. \end{array}$$ (27)

• **Exact LRT p-value:**

Calculations follow as for the other indices and in this scenario

$$\begin{array}{c} h\left(x\right)=\frac{n!2^{x_{2}}\left(2x_{1}+x_{2}\right)!\left(2x_{3}+x_{2}\right)!}{x_{1}!x_{2}!x_{3}!\left(2n+1\right)!}. \end{array}$$ (28)

• **Barnard’s Exact Test:**

The critical region is ***R*** = {λ(***X***) ≤ λ(***x***)}, and the Barnard’s exact p-value is obtained by

$$\begin{array}{c} \text{p-value}=\underset{0\leq \theta \leq 1}{\text{max}}\sum_{R}[\frac{n!}{x_{1}!x_{2}!x_{3}!}]2^{x_{2}}\theta^{2x_{1}+x_{2}}{\left(1-\theta \right)}^{2x_{3}+x_{2}}. \end{array}$$ (29)

• **FBST:**

Assuming a Dirichlet(1, 1, 1) prior for ***θ*** and that *X* follows a Trinomial(*n*, *θ*_1_, *θ*_2_, *θ*_3_) distribution, the posterior distribution is ***θ*** ∣ ***x*** ~ Dirichlet(*x*_1_ + 1, *x*_2_ + 1, *x*_3_ + 1). In this setting,

$$\begin{array}{c} \underset{\theta \in \Theta_{H}}{\text{sup}}\pi \left(\theta |x\right)=\frac{\left(n+2\right)!}{x_{1}!x_{2}!x_{3}!}2^{x_{2}}{\left(\frac{2x_{1}+x_{2}}{2n}\right)}^{2x_{1}+x_{2}}{\left(\frac{x_{2}+2x_{3}}{2n}\right)}^{x_{2}+2x_{3}}. \end{array}$$ (30)

• **Other indices:**

Both asymptotic LRT p-value and asymptotic e-value are obtained, the p-value considering that −2 ln(λ(***X***)) follows a $X^{2}$ distribution with 1 degrees of freedom and the FBST considering that it follows a $X^{2}$ distribution with 2 degrees of freedom.

## 2 Results

### 2.1 Relations between the indices

In many practical situations, mainly in biological studies, asymptotic distributions are used to evaluate indices even for small samples. With that in mind, one of our interests is to understand how the use of asymptotic results for small sample size settings compares to the use of an exact index. Surprisingly, the values of exact and asymptotic indexes do not diverge considerably.

As our objective is to compare the indices, we consider different scenarios for each hypothesis. For each scenario, we evaluate the significance indices of all test procedures presented here. Note that this is not a simulation study; for each sample size, we evaluate the indices for all possible contingency tables of a fixed dimension and size. For example, considering homogeneity hypothesis in a 2 × 2 table with marginals (10, 10), there are 121 possible tables or considering independence hypothesis in a 2 × 3 table with marginal 15, there are 15504 possible tables. We evaluated the indices for all tables that fit into each specification. For the e-value computation, non-informative priors for the parameters are considered (that is, *π*(***θ***) ∝ 1). This way, no extra information is added besides the data, allowing fair comparisons between frequentist and Bayesian indices.

For each scenario, plots are drawn to illustrate differences between the indices’ values. The indices studied are the exact LRT p-value, asymptotic p-value for the LRT, asymptotic p-value for the chi-square test, e-value and asymptotic e-value. For the homogeneity hypothesis in 2 × 2 tables, Fisher and Barnard exact tests were also considered, and for Hardy-Weinberg equilibrium hypothesis the Barnard’s exact test was also obtained. We considered many different scenarios, however, since the aim is to understand the indices in small sample size, the scenarios presented here are in Table 4.

**Table 4:** Considered scenarios.

Setting	Hypothesis	Table	Sample sizes
1	Homogeneity	2 × 2	(30, 30)
2	Homogeneity	2 × 2	(100, 100)
3	Homogeneity	2 × 3	(30, 30)
4	Homogeneity	3 × 3	(15, 15, 15)
5	Independence	2 × 2	30
6	Independence	2 × 3	30
7	Independence	3 × 3	15
8	Independence	3 × 3	25
9	Hardy-Weinberg equilibrium	-	30
10	Hardy-Weinberg equilibrium	-	100

**Fig 3:**
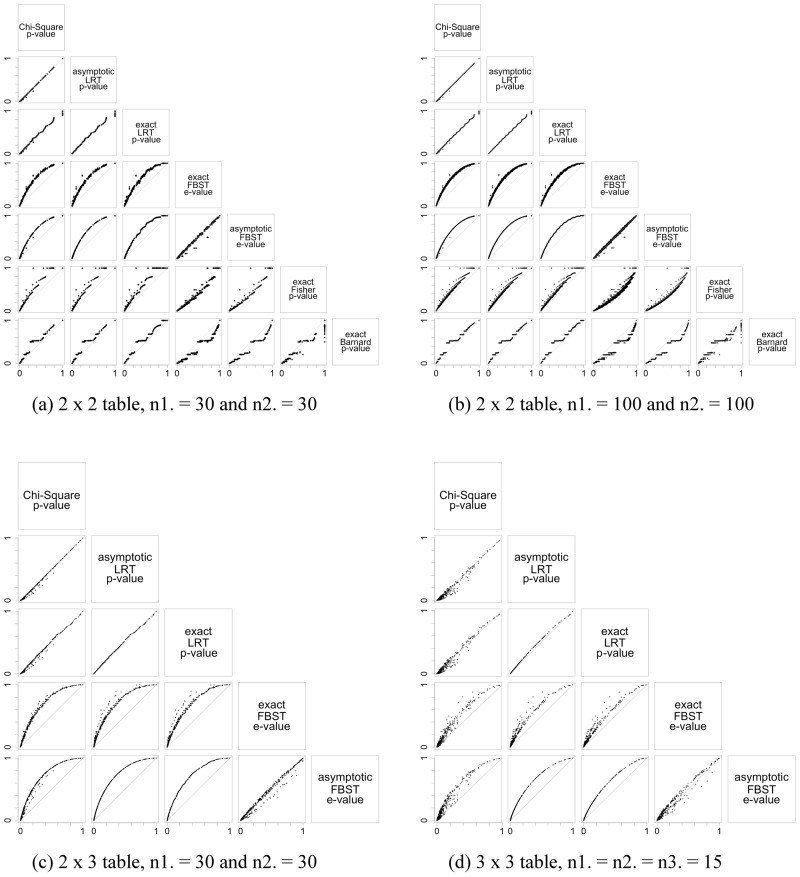
Scaterplots for the significance indices of homogeneity hypothesis considering different sample sizes and different table dimensions. The indices were evaluated for all possible samples in the sample space. The label in the top box of that column give the index in the x-axis, and the label in the left box of that row give the index in the y-axis. Each table dimesions and sample sizes are given in the sublabels.

**Fig 4:**
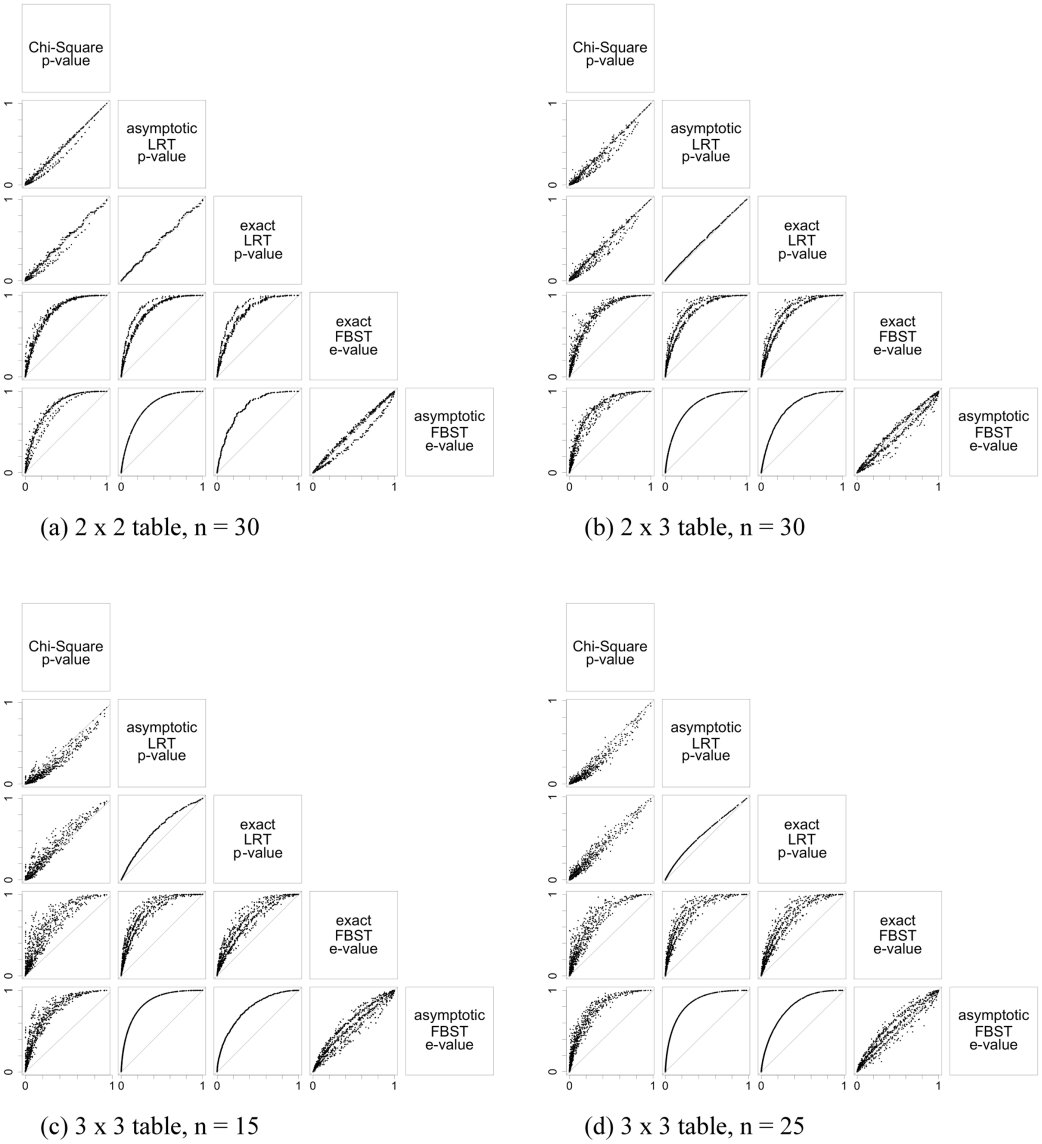
Scaterplots for the significance indices of independence hypothesis considering different sample sizes and different table dimensions. The indices were evaluated for all possible samples in the sample space. The label in the top box of that column give the index in the x-axis, and the label in the left box of that row give the index in the y-axis. Each table dimesions and sample sizes are given in the sublabels.

**Fig 5:**
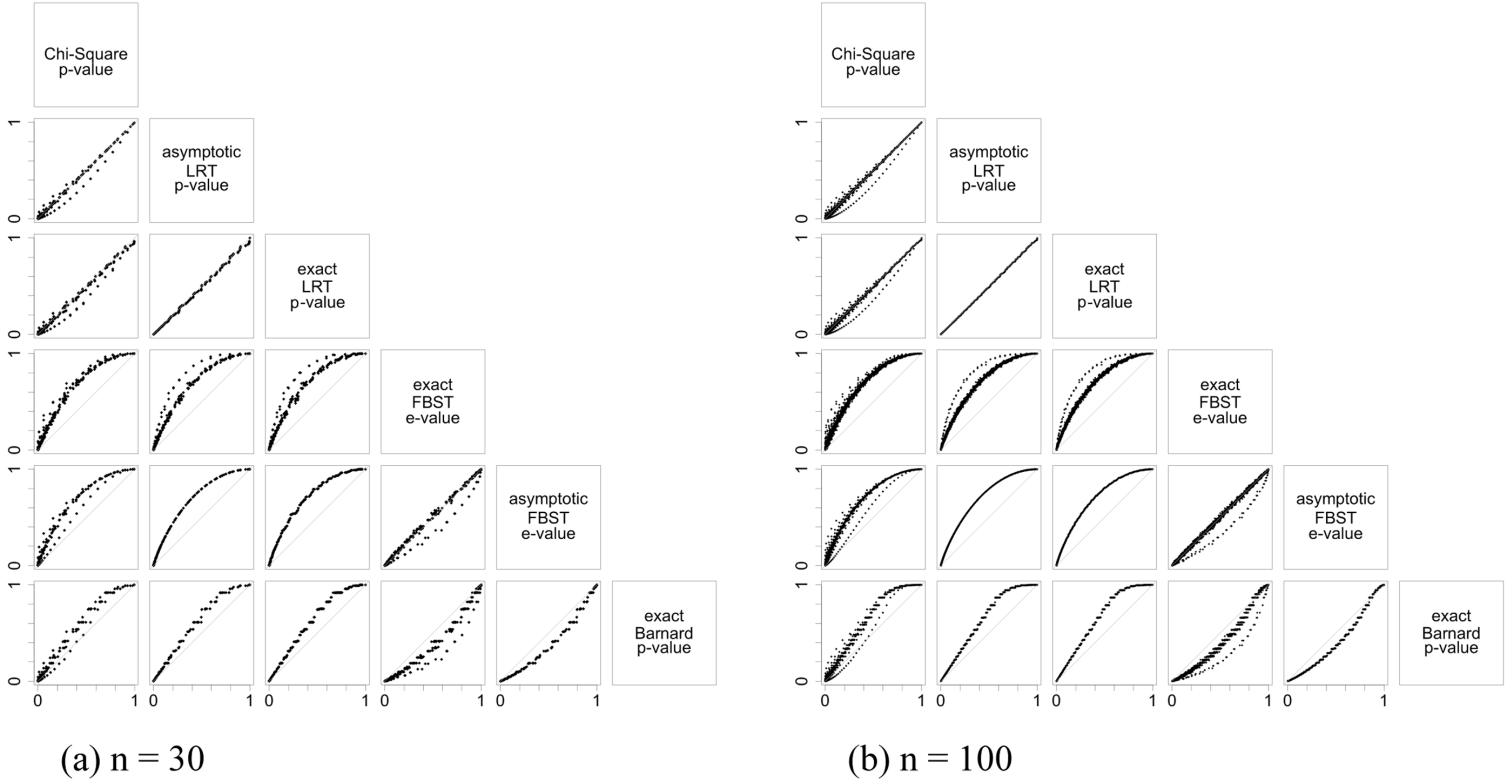
Scaterplots for the significance indices of Hardy-Weinberg hypothesis considering different sample sizes and different table dimensions. The indices were evaluated for all possible samples in the sample space. The label in the top box of that column give the index in the x-axis, and the label in the left box of that row give the index in the y-axis. Each table dimesions and sample sizes are given in the sublabels.

Figs 3, 4 and 5 illustrate the results of the discussion above. For all hypotheses, exact and asymptotic e-values are very similar for both large and small sample sizes. Looking into the frequentist indices, exact LRT p-values and asymptotic p-values, both LRT and Chi-Square, are also very similar to each other. The difference found between e-values when compared to asymptotic LRT p-value happens as a result of the way these indices are formulated: while e-values consider the full dimension of the parameter space, p-value consider the complementary dimension of the set corresponding to hypothesis ***H***. This is expected from the asymptotic relationship between e-value and p-value from the LRT [13, 14]. Since the exact LRT p-value is directly related to the asymptotic LRT p-value, we observe the same behavior of the differences between e-values and asymptotic LRT p-value. Fisher’s exact test was only calculated for the homogeneity hypothesis in 2 × 2 tables, and Barnard’s exact test was calculated for the homogeneity hypothesis in 2 × 2 tables and for the Hardy-Weinberg equilibrium hypothesis. Both indices have a different behavior among the other indices considered. They have a discrete behavior, which is not surprising since Fisher’s exact test is a conditional test and Barnard’s exact test takes a maximization nuisance parameter elimination. Looking at the plots, their values do not form a continuous curve like the other indices’ values do, and its points are quite far from all the other indices.

### 2.2 Power function

Power functions are a useful tool to compare hypothesis tests. For all *θ* ∈ Θ, the power function provides the probability of rejecting the hypothesis for a given *θ*. In fact, we look for a test that does not reject the hypothesis for *θ* ∈ Θ_***H***_ and the further the *θ* value is from the hypothesis, the probability of rejection increases.

The power functions presented are the ones that we are able to represent in ℝ^3^, which are the power functions for the homogeneity hypothesis in 2 × 2 contingency tables and for the Hardy-Weinberg equilibrium hypothesis.

We used p-values less than 0.05 as a decision rule to reject the hypothesis. This choice is based on what is vastly used in most fields of science as a decision rule. In this case, Power(*θ*_1_, *θ*_2_) = P(reject ***H***|(*θ*_1_, *θ*_2_) and Reject ***H*** if index ≤ 0.05.

We obtain the power function for all tests but the FBST. The FBST is a Bayesian significance test and in order to obtain a power function, one would need a decision rule. Since its construction differs from that of the p-values, we cannot use the same decision rule, and constructing a decision rule is not in the scope of this paper.

We used a Monte Carlo procedure to evaluate the power function of these tests. We consider a grid for the unit square with 100 × 100 points on the axes (*θ*_1_, *θ*_2_). For each point in the grid we generated 1000 tables. From these 1000 tables we evaluate the proportion of rejections, which is an approximation of the power function.

**Fig 6:**
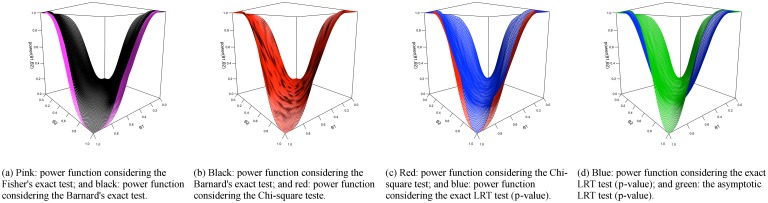
Power function for homogeneity hypothesis in 2 × 2 contingency tables with *n*_1⋅_ = *n*_2⋅_ = 10.

**Fig 7:**
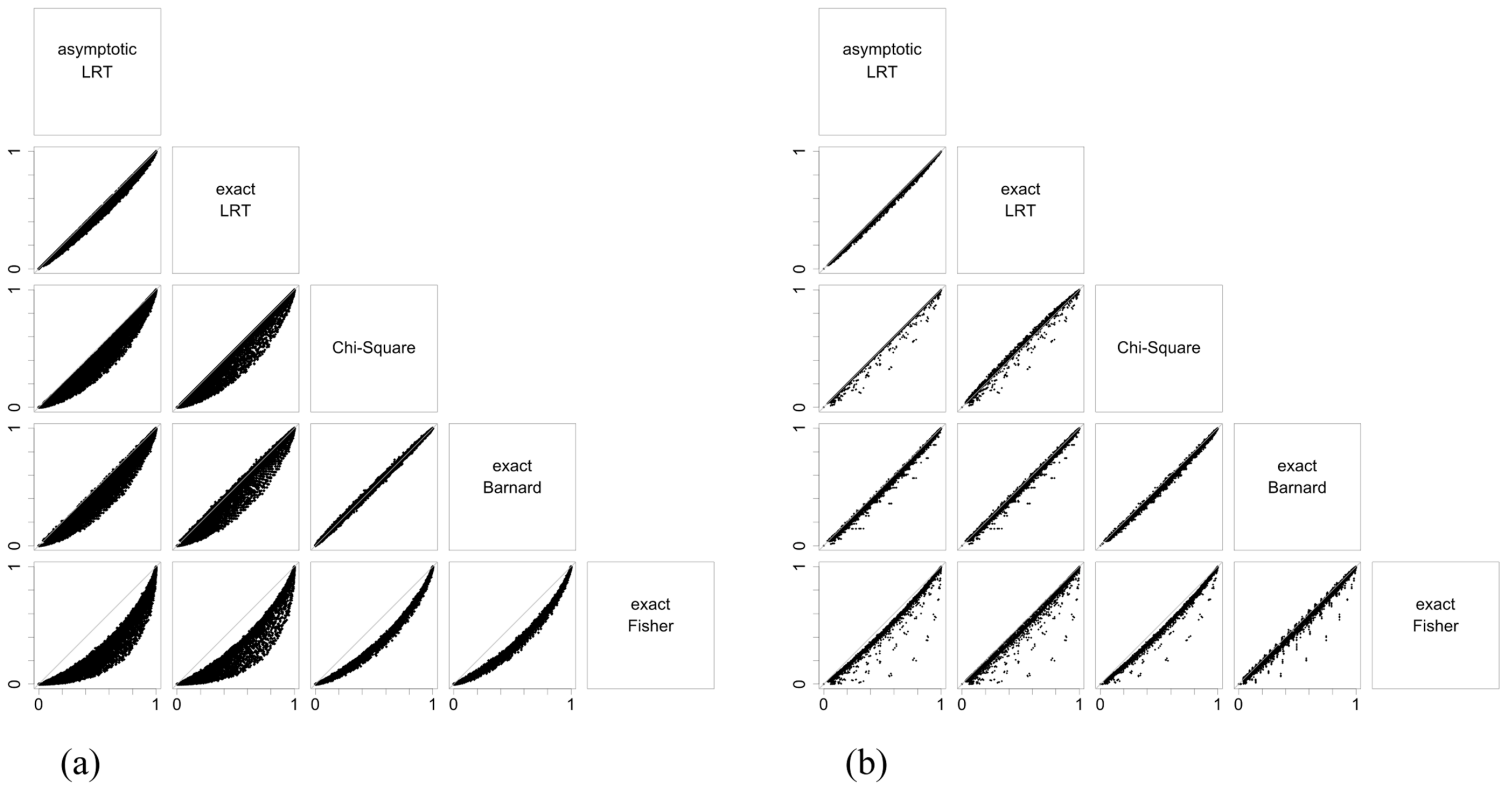
Plots of power function values for the homogeneity test. Each graph presents one index versus another, each dot representing a point in the considered parametric space (in this case, 100 × 100 = 10000 points), and if a dot is on top of the gray identity line, the power functions assume the same value for that point in the parametric space. The scenario is 2 × 2 with marginals *n*_1⋅_ = *n*_2⋅_ = 10 in (a) and *n*_1⋅_ = *n*_2⋅_ = 100 in (b).

We plot pairs of power functions to illustrate and compare their shapes. For the homogeneity hypothesis in a table with marginals (10, 10), Fig 6 shows that Fisher’s exact test is less powerful than the Barnard’s exact test, the Barnard’s exact test is has similar power when compared with the Chi-square test, while the Chi-square is less powerful than the proposed exact LRT p-value, which is less powerful than the asymptotic p-value for the LRT. To have a clear picture, we plot the power functions from different tests against each other. Fig 7a consists of the power functions for tables with marginal equals to (10, 10). It shows that the use of the asymptotic p-value for the LRT results in a more powerful test than the other indices. When comparing the proposed exact p-value to other indices, it is more powerful than the Chi-square test and the Fisher’s exact test. Between the Chi-square and the Fisher’s exact test, the Chi-square test is more powerful.

For tables with marginal equals to (100, 100), the graphs are more concentrated near the identity line (Fig 7b), showing that all indices are more alike. The ordering still exists, but it is less severe. It is interesting to point out that, as expected, the Chi-square test works better with larger samples.

**Fig 8:**
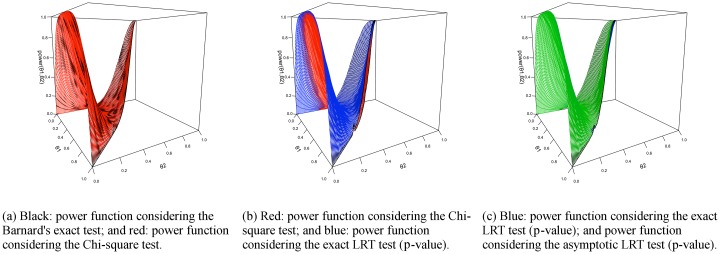
Power function for Hardy-Weinberg equilibrium hypothesis with *n* = 10.

**Fig 9:**
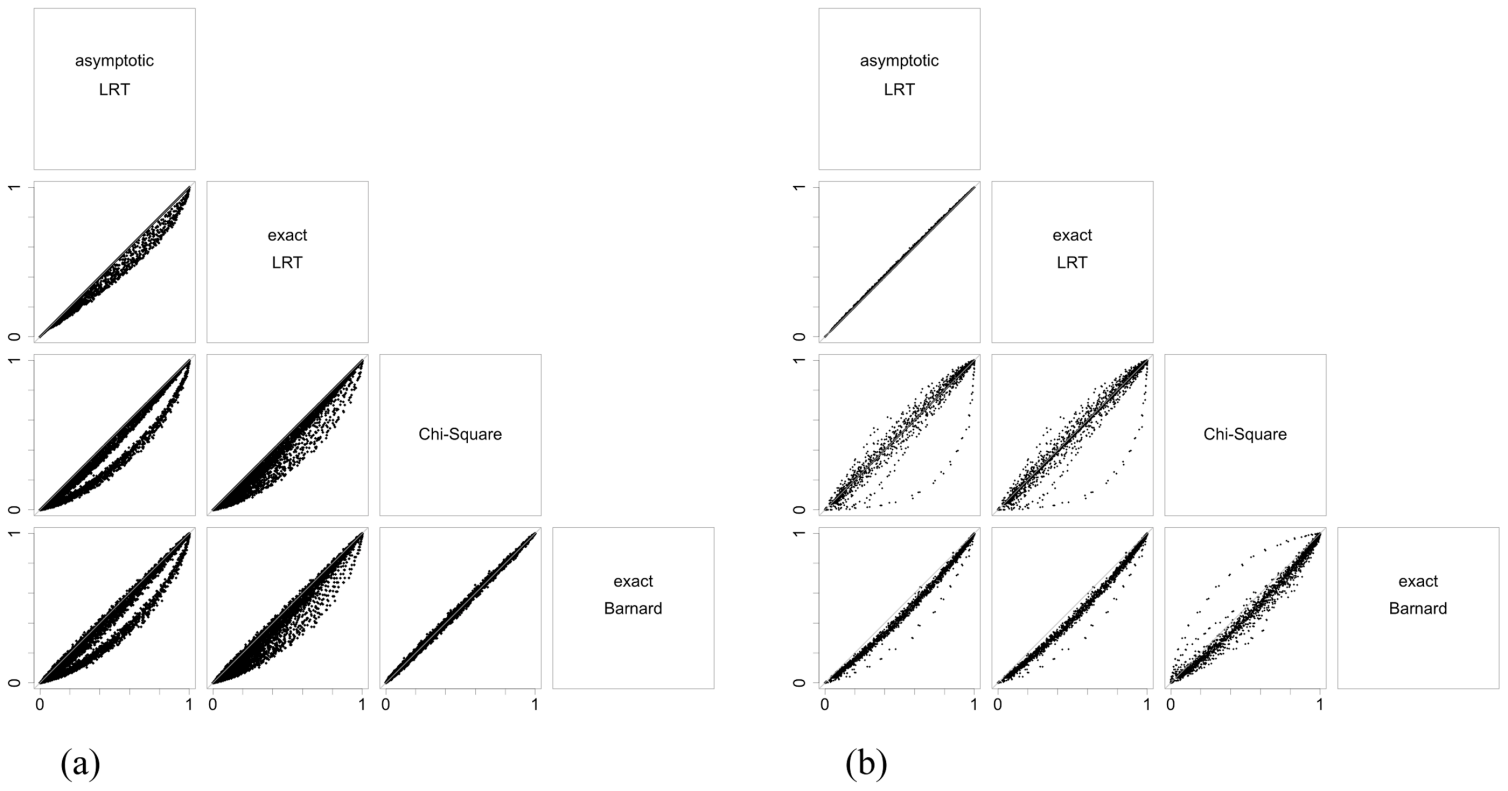
Plots of power functions values for the Hardy-Weinberg equilibrium test. Each graph presents one index versus another, each dot representing a point in the considered parametric space (in this case, 100 × 100 = 10000 points), and if a dot is on top of the gray identity line, the power functions assume the same value for that point in the parametric space. The scenarios are marginals *n* = 10 (a) and *n* = 100 (b).

For the Hardy-Weinberg hypothesis, the results are similar to the ones obtained for the homogeneity hypothesis and are shown in Figs 8 and 9. In this case, the most powerful test was the asymptotic p-value for the LRT, followed by the exact p-value for the LRT, which is more powerful to the Chi-square test, that is similar the Barnard’s exact test. We call attention to the fact that, under hypothesis ***H***, the power function achieves the value of 0.05, as expected, since this is the significance level chosen to build the power functions.

## 3 Conclusion

After evaluating the indices for tables in different scenarios, we noticed that all of them had very similar behaviors, independently of the perspective (Bayesian or frequentist), sample size and table dimension. The exceptions are the p-values for Fisher and Barnard’s exact tests for the homogeneity hypothesis in 2 × 2 tables, and Barnard’s exact test for Hardy-Weinberg equilibrium, which show a discretized behavior. Studying the power functions considering homogeneity hypothesis in 2 × 2 tables and Hardy-Weinberg equilibrium hypothesis, the LRT presented itself as a powerful test when considering small sample sizes, while Fisher’s exact test was the least powerful one for the homogeneity hypothesis and the Barnard’s exact test was the least powerful for the Hardy-Weinberg equilibrium hypothesis. By enlarging sample sizes, the power of these tests increases accordingly.

Finally, we finish this paper listing our main conclusions:

- The LTR asymptotic p-value seems to be a good frequentist alternative for small sample sizes.
- Since there is an asymptotic relationship between the p-value for the LRT and the e-value (FBST), we consider that both indices are equivalent in the explored settings.
- In cases where there is available information besides the data that to be taken into account, represented by informative priors, we consider the e-value a more appropriate index than a frequenstist one, since the e-value offers a mechanism to incorporate that information.
